# Early prediction of incident delirium in traumatic brain injury: a multicenter validated and interpretable machine learning approach

**DOI:** 10.3389/fneur.2026.1848730

**Published:** 2026-05-21

**Authors:** Cheng Li, Tianyi Zhang, Hong Chen, Shouli Wang, Lei Wang, Yixiang Huan, Jianchao Liu, Lihua Liu

**Affiliations:** 1 Graduate School of PLA General Hospital, PLA General Hospital, Beijing, China; 2 Department of Medical Innovation and Research, PLA General Hospital, Beijing, China; 3 Graduate School of Capital Medical University, Capital Medical University, Beijing, China; 4 Department of Neurosurgery, Beijing Fangshan District Liangxiang Hospital, Beijing, China; 5 Department of Medical Psychology, Ninth Medical Center of PLA General Hospital, Beijing, China

**Keywords:** delirium, machine learning, neuropsychiatric complications, random forest model, traumatic brain injury

## Abstract

**Objective:**

This study aims to develop and externally evaluate a machine learning (ML)-based predictive model for incident delirium in patients with traumatic brain injury (TBI).

**Methods:**

Patients diagnosed with TBI from the MIMIC-IV and eICU-CRD databases were included. Predictors were selected using Boruta and LASSO regression. Five ML algorithms were developed and compared, with logistic recalibration applied to the external cohort. Model performance was assessed using the area under the receiver operating characteristic curve (AUC), calibration curves, and decision curve analysis (DCA). Shapley Additive Explanations (SHAP) was utilized to decode individual risk contributions. Subgroup and sensitivity analyses were conducted to define clinical boundaries and evaluate model robustness.

**Results:**

A total of 915 TBI patients from the MIMIC-IV database and 317 from the eICU-CRD database were included. Random Forest (RF) model achieved balanced performance with an internal AUC of 0.819 and an external AUC of 0.706. The model exhibited favorable internal calibration, adequate external recalibration, and positive clinical net benefits (internal: 0.155, external: 0.080). Overall SHAP analysis identified invasive ventilation, Glasgow Coma Scale (GCS), extracranial injury, Acute Physiology Score III (APSIII), hemoglobin and mixed intra-/extra-axial injury as primary predictors. Crucially, stratified SHAP analysis identified invasive ventilation as the primary driver across all strata, with baseline GCS scores attaining their maximum predictive weight in the medium-risk tier. Subgroup analyses of the external cohort indicated robust generalization in younger patients (AUC = 0.780) and those with extracranial injuries (AUC = 0.762), with expected attenuation in subgroups with higher clinical severity (AUC: 0.578–0.589). Sensitivity analyses confirmed the model's stable performance against competing mortality and missing data (all DeLong test *p* > 0.05).

**Conclusions:**

The RF model demonstrated acceptable discriminative capacity and clinical utility for early delirium prediction in patients with TBI. Supported by SHAP, it translated complex predictions into an actionable three-tiered framework, serving as a valuable adjunct for guiding early monitoring and neuroprotective strategies.

## 1 Introduction

Traumatic brain injury (TBI) is a severe and often life-threatening emergency requiring intensive monitoring and care (1). Patients with TBI are particularly susceptible to developing delirium, which is characterized by acute onset, fluctuating consciousness, and significant cognitive dysfunction (2). Reported incidence rates of delirium in this high-risk population range from 32.4% to 60.0% (3–6). Unlike general ICU delirium, delirium in TBI patients is driven by distinct pathophysiological mechanisms, including the primary brain injury and subsequent neuroinflammatory cascades that severely disrupt neurotransmitter homeostasis (3). The onset of delirium is independently associated with increased mortality and long-term cognitive impairment, significantly impeding neuropsychiatric functional recovery and diminishing overall quality of life (7, 8).

Despite the clinical significance of this condition, accurate risk stratification remains a challenge. In current clinical practice, the Prediction of Delirium in ICU patients (PRE-DELIRIC) model, grounded in traditional logistic regression (LR), is widely utilized as the standard tool for predicting delirium (9, 10). However, as a generalized tool developed for a broad ICU cohort, PRE-DELIRIC lacks disease-specific variables, which may result in suboptimal performance to fully capture unique risk factors when applied to specific patient populations (11–13). Furthermore, to the best of our knowledge, there are currently no validated prediction models designed specifically for early delirium prediction in the TBI population.

Recently, machine learning (ML) has emerged as a transformative approach in medical risk prediction, offering the ability to process high dimensional datasets and capture non-linear interactions among diverse clinical variables (14, 15). While emerging evidence suggests that ML provides robust frameworks for delirium prediction in general settings (16–18), its full potential has yet to be explored and validated in the TBI subgroup.

Consequently, this study seeks to develop and externally evaluate a machine learning model for early delirium prediction in TBI patients. Beyond initial discriminative performance, we prioritize model calibration via external recalibration to improve its applicability across diverse clinical settings, aiming to provide an adjunctive tool to inform early neuroprotective strategies.

## 2 Methods

### 2.1 Data source

The training set and internal test set for this study were derived from the MIMIC-IV v3.1 database, which included 94,458 ICU stay records collected from 2012 to 2022 at the Beth Israel Deaconess Medical Center in the United States (19). External validation data were obtained from the eICU-CRD v2.0, a multicenter ICU database comprising 200,859 ICU stay records from 335 ICUs across 205 hospitals in the United States between 2014 and 2015 (20). The author Li Cheng completed the Collaborative Institutional Training Initiative (CITI) program and obtained certification (Record ID: 73397179).

The MIMIC-IV database and the eICU-CRD have been approved for use by the institutional review boards of Beth Israel Deaconess Medical Center and Massachusetts Institute of Technology, in accordance with the Declaration of Helsinki. Both databases received waivers for informed consent due to the deidentification of all protected health information.

### 2.2 Study population

**Figure 1:**
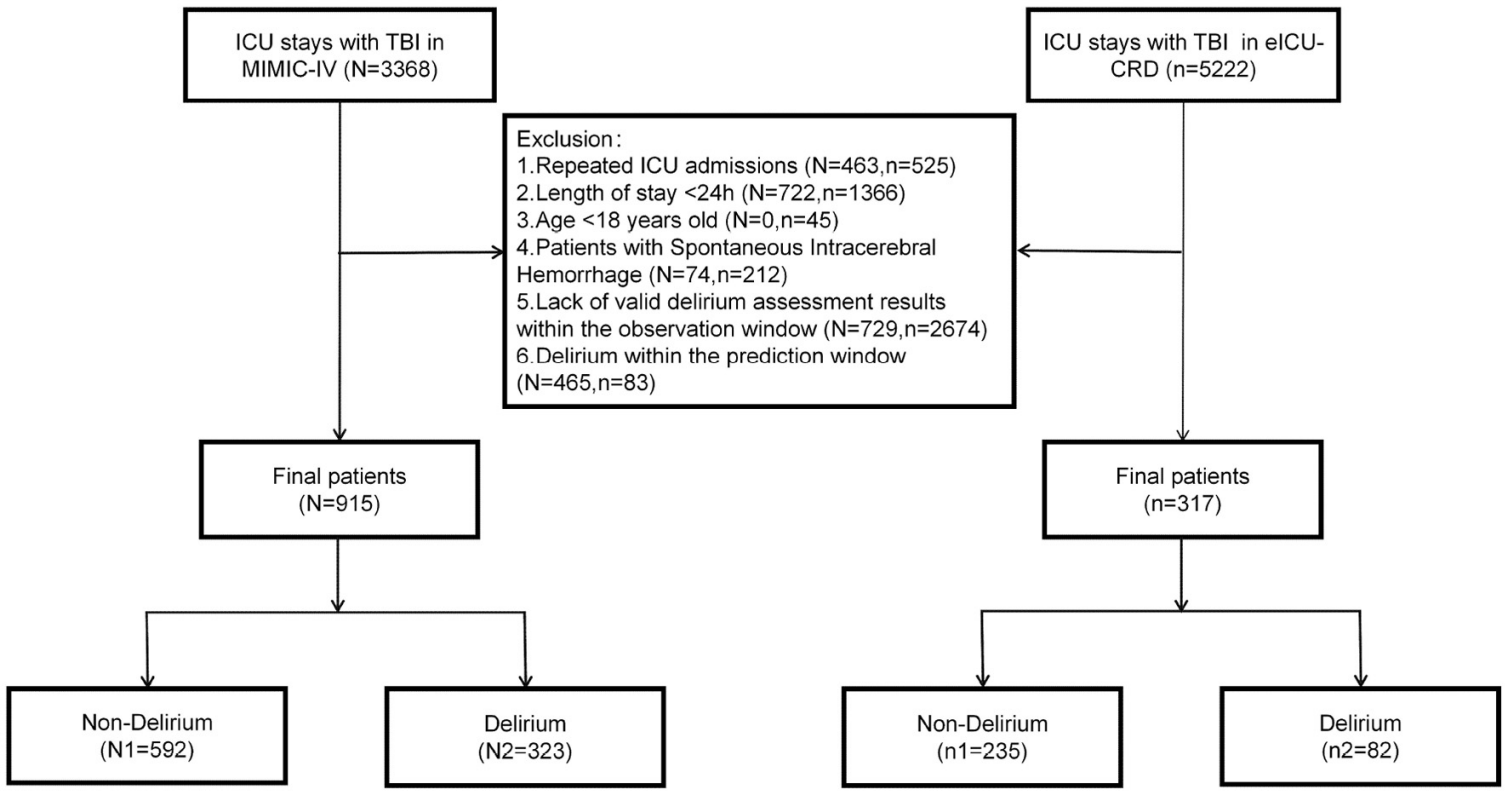
Flow chart of this study.

Patients with TBI were identified based on the International Classification of Diseases, Ninth and Tenth Revisions (ICD). Specifically, we included ICU-admitted patients with ICD-9 codes starting with 851, 852, 853, or 854, and ICD-10 codes starting with S06.1, S06.2, S06.3, S06.4, S06.5, S06.6, or S06.8. The exclusion criteria were: (1) Repeated ICU admissions (only data from the first ICU admission were included); (2) ICU length of stay < 24 h; (3) age < 18 years old; (4) patients with spontaneous intracerebral hemorrhage (sICH); (5) lack of valid delirium assessment results within the observation window; and (6) onset of delirium during the prediction window. Furthermore, to maintain consistency with the MIMIC-IV database, the ages of patients >89 years old in the eICU database were uniformly converted to 91. The prediction window was designated as the first 24 h following ICU admission, and the observation window was defined as the period starting from the second day of ICU admission until ICU discharge or death. The detailed screening process was illustrated in Figure 1.

### 2.3 Data extraction

Data extracted for this study included: (1) demographics information: age, gender, height, weight; (2) trauma characteristics: types of TBI (One-hot encoding), skull fractures, extracranial injuries; (3) vital signs and laboratory results within the first 24 h of ICU admission. (4) comorbidities: dementia, stroke, Parkinson, major psychotic dsorder, depression, alcohol abuse, nicotine dependence, Malnutrition, hypertension, diabetes, coronary heart disease, congestive heart failure, COPD, chronic kidney disease, chronic liver disease and tumor; (5) disease severity scores: Glasgow Coma Scale (GCS) score, Acute Physiology Score III (APSIII), and Sequential Organ Failure Assessment (SOFA) score; and (6) interventions within the first 24 h of ICU admission: neurosurgical intervention, invasive ventilation, blood transfusion, and administration of specific drugs including benzodiazepines, propofol, dexmedetomidine, opioids, steroids, mannitol, vasopressors, antihypertensives, acetaminophen, non-steroidal anti-inflammatory drugs (NSAIDs), insulin and vitamin K. For variables with multiple measurements, the worst values recorded within the initial 24 h were selected to capture the peak physiological derangement.

The primary outcome was the incidence of delirium within the prediction window. Delirium was defined as a positive screen on the Confusion Assessment Method for the ICU (CAM-ICU) or an Intensive Care Delirium Screening Checklist (ICDSC) score of 4 or greater (21).

### 2.4 Data processing

Data extraction and preprocessing were conducted in R using the *RPostgres* and *dbplyr* packages to query the local PostgreSQL database. Prior to imputation, implausible physiological outliers were converted to missing values (22). Variables with missing values exceeding 25%, namely temperature, height, arterial partial pressure of oxygen (PaO_2_), pH, partial thromboplastin time (PTT), alanine aminotransferase (ALT), and aspartate aminotransferase (AST), were excluded from the analysis, as forceful imputation of these highly incomplete features would introduce unacceptable noise into the model. The remaining missing data were imputed using a non-parametric random forest algorithm via the *missForest* package (ntree = 500), as it accounts for non-linear interactions and multicollinearity without requiring strict distributional assumptions, which is consistent with the applied machine learning framework (23).

### 2.5 Feature selection

Relevant features were first screened via the Boruta algorithm, which operates by creating “shadow features” and training a Random Forest classifier (21). Attributes with importance significantly higher than the shadow features were deemed confirmed, while tentative attributes were resolved using a rough fix comparison. We then refined the feature set using LASSO regression with 10-fold cross-validation (24). To strictly control model complexity, we selected the penalty parameter based on the 1-standard-error rule (lambda 1se). Only features retaining non-zero coefficients at this threshold were included in the final model.

### 2.6 Model development and validation

The dataset was randomly partitioned into a training set and a testing set in a 7:3 ratio using the rsample” package. Stratified sampling was performed based on the outcome variable to preserve the class distribution across both sets. A random seed of 123 was applied to ensure the reproducibility of the data splitting. In total, 5 algorithms, namely logistic regression (LR), elastic net regression (EN), K-nearest neighbors (KNN), random forest (RF) and extreme gradient boosting (XGBoost) were developed.

For the models developed within the caret framework (LR, EN, KNN, and RF), hyperparameters were optimized using 10-fold cross-validation via a grid search strategy. In contrast, the XGBoost model was independently optimized using a native implementation with 80 random search iterations. Each iteration utilized 10-fold cross-validation with an early stopping mechanism to prevent overfitting and determine the optimal boosting rounds. To address potential heterogeneity in baseline delirium prevalence and case-mix within the external validation cohort, a logistic recalibration strategy was implemented to refine risk estimates (25). The parameters of model development and validation were reported in Supplementary Table 1. Model performance was assessed using the Area Under the Receiver Operating Characteristic curve (AUC), accuracy, sensitivity, specificity, Positive Predictive Value (PPV) and Negative Predictive Value (NPV). Calibration was evaluated via calibration curves, the integrated calibration index (ICI), and Brier scores. Clinical utility was determined through decision curve analysis (DCA).

### 2.7 Model interpretability and clinical explainability

The SHAP (Shapley Additive Explanations) method, grounded in cooperative game theory, was employed to interpret the optimal model (15). This approach quantifies the contribution of each predictor to the final probability estimate of delirium. Specifically, SHAP values were estimated using a Monte Carlo sampling approach with 500 simulations via the fastshap package. For the background dataset, 200 samples were randomly selected from the training set to establish the base expectation (E[f(x)]). To assess overall feature importance and the directionality of predictor impacts across the cohort, global SHAP values were visualized via summary bar charts and beeswarm plots. Furthermore, individual patient contributions for representative cases from the testing set (one patient with delirium and one without) were visualized using force plots and waterfall plots via the *shapviz* package. All quantitative contributions and probabilities were reported to provide a clear and rigorous interpretable framework for the delirium risk prediction model. To further delineate how predictor impacts evolve across different clinical states, a stratified SHAP analysis was performed. Specifically, the testing cohort was partitioned into low-, medium-, and high-risk tiers based on the tertiles of the predicted delirium probabilities. For each tier, the mean absolute SHAP values for the primary predictors were calculated to quantify their respective importance in driving risk within that specific stratum.

### 2.8 Sentitivity and subgroup analysis

To address potential underestimation of delirium due to early mortality, we performed a sensitivity analysis using a validated “worst-case scenario” approach (26, 27). Specifically, deceased patients without prior delirium were reclassified as delirium-positive to ensure model robustness against survivorship bias. Additionally, to validate our missing data strategy, we performed a sensitivity analysis using Multiple Imputation by Chained Equations (MICE; 5 imputations/iterations) with predictive mean matching. Model performance was evaluated by averaging predicted probabilities across models independently trained on each dataset, with final metrics pooled via Rubin's Rules (28). Finally, to evaluate model robustness and potential performance heterogeneity across diverse clinical subpopulations, SHAP-guided subgroup analyses were performed in both cohorts.

### 2.9 Statistical analysis

As all continuous variables exhibited non-normal distributions, they were summa–Whitney *U* tests, while categorical variables were presented as frequencies (percentages) and analyzed using Chi-square or Fisher's exact tests. All statistical analyses were conducted using R version 4.5.0 (R Foundation for Statistical Computing, Vienna, Austria) and *p* < 0.05 was considered statistically significant.

## 3 Results

### 3.1 Baseline characteristics

The final cohort included 915 patients from MIMIC-IV and 317 from eICU-CRD, with incident delirium identified in 323 (35.30%) and 82 (25.87%) patients, respectively. Table 1 presents the characteristics of patients who had delirium and those who did not within the observation window.

**Table 1:** Baseline characteristics of patients with and those without delirium in the observation window.

Patients characteristics	MIMIC-IV		*P* value	eICU-CRD		*P* value
	No delirium (*n* = 592)	Delirium (*n* = 323)		No delirium (*n* = 235)	Delirium (*n* = 82)	
Demographic data						
Age (years), median (IQR)	70.00 (57.00, 81.00)	67.00 (51.00, 81.00)	0.036	63.00 (42.00, 81.00)	62.50 (47.00, 80.00)	0.824
Male gender, *n* (%)	361 (60.98%)	211 (65.33%)	0.199	136 (57.87%)	54 (65.85%)	0.239
Weight (kg), median (IQR)	75.45 (64.35, 86.85)	75.60 (62.40, 88.00)	0.739	76.80 (67.00, 90.00)	77.10 (65.70, 94.00)	0.762
Trauma characteristics						
TBI types						
Extra-axial Injury, *n* (%)	476 (79.90%)	205 (63.47%)	<0.001	110 (46.81%)	40 (48.48%)	0.858
Intra-axial Injury, *n* (%)	72 (12.16%)	51 (15.79%)	0.129	97 (41.28%)	25 (30.49%)	0.088
Mixed Injury, *n* (%)	47 (7.94%)	67 (20.74%)	<0.001	28 (11.91%)	17 (20.73%)	0.065
Skull fracture, *n* (%)	75 (12.67%)	75 (23.22%)	<0.001	21 (8.94%)	12 (14.63%)	0.147
Extracranial injury, *n* (%)	187 (31.59%)	178 (55.11%)	<0.001	61 (25.96%)	23 (28.05%)	0.772
Vital signs, median (IQR)						
Heart rate, (beats/min)	95.00 (83.00, 108.00)	102.00 (90.00, 120.00)	<0.001	103.00 (87.00, 120.00)	109.50 (93.00, 125.00)	0.148
Respiratory rate, (beats/min)	25.00 (22.00, 28.00)	26.00 (23.00, 30.00)	0.002	33.00 (27.00, 40.00)	29.00 (25.00, 34.00)	0.008
Spo2, (%)	93.00 (91.00, 95.00)	94.00 (92.00, 96.00)	<0.001	90.00 (86.00, 94.00)	91.00 (84.00, 94.00)	0.680
SBP, (mm Hg)	151.00 (139.00, 163.00)	156.00 (142.00, 170.00)	0.006	161.00 (136.00, 178.00)	171.00 (152.00, 183.00)	0.018
DBP, (mm Hg)	54.00 (45.00, 98.00)	52.00 (43.00, 96.00)	0.192	54.00 (44.00, 99.00)	87.00 (50.00, 109.00)	0.009
MBP, (mm Hg)	104.00 (62.00, 115.00)	103.00 (60.00, 118.00)	0.888	111.00 (63.00, 128.00)	119.00 (102.00, 130.00)	0.077
Laboratory results, median (IQR)						
White blood cells, (109/L)	10.20 (7.60, 13.50)	12.70 (9.50, 16.60)	<0.001	9.80 (8.10, 12.70)	11.05 (8.60, 13.60)	0.127
Hemoglobin, (g/dL)	11.70 (10.10, 12.70)	10.40 (9.00, 11.70)	<0.001	11.80 (10.50, 12.80)	11.45 (10.00, 12.60)	0.273
Hematocrit, (%)	35.10 (31.00, 38.30)	31.70 (27.40, 35.20)	<0.001	35.50 (31.70, 38.30)	34.50 (30.60, 38.20)	0.250
Platelets, (109/L)	184.00 (143.00, 227.00)	161.00 (122.00, 200.00)	<0.001	199.00 (149.00, 236.00)	181.50 (138.00, 230.00)	0.107
Sodium, (mmol/L)	138.00 (136.00, 142.00)	139.00 (136.00, 144.00)	0.062	139.00 (137.00, 141.00)	140.00 (137.00, 142.00)	0.140
Potassium, (mmol/L)	3.90 (3.60, 4.30)	3.90 (3.50, 4.20)	0.308	3.90 (3.60, 4.20)	3.90 (3.50, 4.10)	0.160
Calcium, (mg/dL)	8.60 (8.20, 8.95)	8.20 (7.70, 8.70)	<0.001	8.60 (8.30, 8.80)	8.30 (7.90, 8.80)	0.045
Chloride, (mmol/L)	105.00 (101.00, 107.00)	106.00 (102.00, 110.00)	<0.001	105.00 (102.00, 108.00)	105.00 (102.00, 108.00)	0.679
Bicarbonate, (mmol/L)	23.00 (21.00, 24.00)	21.00 (19.00, 24.00)	<0.001	24.00 (22.00, 26.00)	24.00 (21.00, 26.00)	0.558
Blood urea nitrogen, (mg/dL)	17.00 (12.00, 23.00)	17.00 (13.00, 23.00)	0.053	14.00 (11.00, 20.00)	16.00 (11.00, 22.00)	0.350
Creatinine, (mg/dL)	0.90 (0.70, 1.10)	0.90 (0.70, 1.20)	0.035	0.88 (0.70, 1.08)	0.87 (0.67, 1.13)	0.877
Glucose, (mg/dL)	126.00 (96.00, 152.00)	147.00 (117.00, 184.00)	<0.001	124.00 (100.00, 152.00)	132.00 (114.00, 153.00)	0.052
INR	1.20 (1.10, 1.30)	1.20 (1.10, 1.40)	0.008	1.15 (1.10, 1.22)	1.19 (1.10, 1.28)	0.100
Comorbidity, *n* (%)						
Dementia, *n* (%)	31 (5.24%)	32 (9.91%)	0.009	18 (7.66%)	7 (8.54%)	0.813
Stroke, *n* (%)	90 (15.20%)	47 (14.55%)	0.846	129 (54.89%)	56 (68.29%)	0.038
Parkinson, *n* (%)	13 (2.20%)	11 (3.41%)	0.285	2 (0.85%)	1 (1.22%)	>0.999
Major psychotic disorder, *n* (%)	23 (3.89%)	17 (5.26%)	0.398	6 (2.55%)	2 (2.44%)	>0.999
Depression, *n* (%)	80 (13.51%)	46 (14.24%)	0.764	23 (9.79%)	4 (4.88%)	0.250
Alcohol abuse, *n* (%)	88 (14.86%)	85 (26.32%)	<0.001	9 (3.83%)	6 (7.32%)	0.228
Nicotine dependence, *n* (%)	174 (29.39%)	79 (24.46%)	0.122	5 (2.13%)	4 (4.88%)	0.244
Malnutrition, *n* (%)	30 (5.07%)	45 (13.93%)	<0.001	11 (4.68%)	9 (10.98%)	0.062
Hypertension, *n* (%)	356 (60.14%)	198 (61.30%)	0.777	157 (66.81%)	57 (69.51%)	0.683
Diabetes, *n* (%)	116 (19.59%)	77 (23.84%)	0.149	52 (22.13%)	16 (19.51%)	0.755
Coronary heart disease, *n* (%)	123 (20.78%)	61 (18.89%)	0.546	54 (22.98%)	25 (30.49%)	0.184
Congestive heart failure, *n* (%)	73 (12.33%)	45 (13.93%)	0.536	19 (8.09%)	7 (8.54%)	>0.999
COPD, *n* (%)	48 (8.11%)	27 (8.36%)	0.900	43 (18.30%)	32 (39.02%)	<0.001
Chronic kidney disease, *n* (%)	60 (10.14%)	42 (13.00%)	0.189	20 (8.51%)	5 (6.10%)	0.636
Chronic liver disease, *n* (%)	31 (5.24%)	24 (7.43%)	0.192	2 (0.85%)	3 (3.66%)	0.111
Tumor, *n* (%)	33 (5.57%)	12 (3.72%)	0.263	7 (2.98%)	4 (4.88%)	0.484
Scores						
GCS, median (IQR)	14.00 (14.00, 15.00)	14.00 (13.00, 15.00)	0.004	14.00 (11.00, 15.00)	10.00 (6.00, 14.00)	<0.001
ASPIII, median (IQR)	30.00 (23.00, 39.00)	37.00 (29.00, 50.00)	<0.001	33.00 (25.00, 50.00)	46.00 (29.00, 64.00)	0.002
SOFA, median (IQR)	2.00 (1.00, 4.00)	3.00 (2.00, 6.00)	<0.001	2.00 (1.00, 4.00)	3.50 (2.00, 5.00)	<0.001
Treatment measures						
Neurosurgical intervention, *n* (%)	179 (30.24%)	90 (27.86%)	0.495	13 (5.53%)	10 (12.20%)	0.080
Invasive ventilation, *n* (%)	106 (17.91%)	188 (58.20%)	<0.001	53 (22.55%)	42 (51.22%)	<0.001
Blood transfusion, *n* (%)	62 (10.47%)	55 (17.03%)	0.005	31 (13.19%)	10 (12.20%)	>0.999
Benzodiazepine, *n* (%)	105 (17.74%)	83 (25.70%)	0.006	59 (25.11%)	32 (39.02%)	0.023
Propofol, *n* (%)	105 (17.74%)	181 (56.04%)	<0.001	44 (18.72%)	34 (41.46%)	<0.001
Dexmedetomidine	15 (2.53%)	35 (10.84%)	<0.001	5 (2.13%)	3 (3.66%)	0.431
Opioids, *n* (%)	304 (51.35%)	213 (65.94%)	<0.001	131 (55.74%)	52 (63.41%)	0.245
Mannitol, *n* (%)	5 (0.84%)	14 (4.33%)	<0.001	2 (0.85%)	4 (4.88%)	0.041
Acetaminophen, *n* (%)	536 (90.54%)	254 (78.64%)	<0.001	150 (63.83%)	51 (62.20%)	0.792
NSAIDs, *n* (%)	2 (0.34%)	3 (0.93%)	0.352	0 (0.00%)	0 (0.00%)	>0.999
Steroids, *n* (%)	34 (5.74%)	16 (4.95%)	0.652	15 (6.38%)	5 (6.10%)	>0.999
Vasopressor, *n* (%)	42 (7.09%)	72 (22.29%)	<0.001	13 (5.53%)	6 (7.32%)	0.591
Antihypertensive, *n* (%)	398 (67.23%)	190 (58.82%)	0.012	110 (46.81%)	40 (48.78%)	0.798
Insulin, *n* (%)	64 (10.81%)	61 (18.89%)	<0.001	60 (25.53%)	28 (34.15%)	0.152
Vitamin K, *n* (%)	55 (9.29%)	34 (10.53%)	0.561	4 (1.70%)	2 (2.44%)	0.651

TBI, traumatic brain injury; SBP, systolic blood pressure; DBP, diastolic blood pressure; MBP, mean blood pressure; INR, international normalized ratio; COPD, chronic obstructive pulmonary disease; APSIII, acute physiology score III; GCS, Glasgow coma scale; SOFA, sequential organ failure assessment; NSAIDs, non-steroidal anti-inflammatory drugs.

### 3.2 Variables for modeling study

Initially, 61 potential influencing factors were included. After screening with the Boruta algorithm, 28 candidate features were retained (Supplementary Figure 1). A subsequent refinement using LASSO regression resulted in a final set of 8 key predictors: invasive ventilation, mixed intra-/extra-axial injury, extracranial injury, dementia, malnutrition, GCS, hemoglobin, APSIII (Supplementary Figure 2).

### 3.3 Model performance evaluation

**Figure 2:**
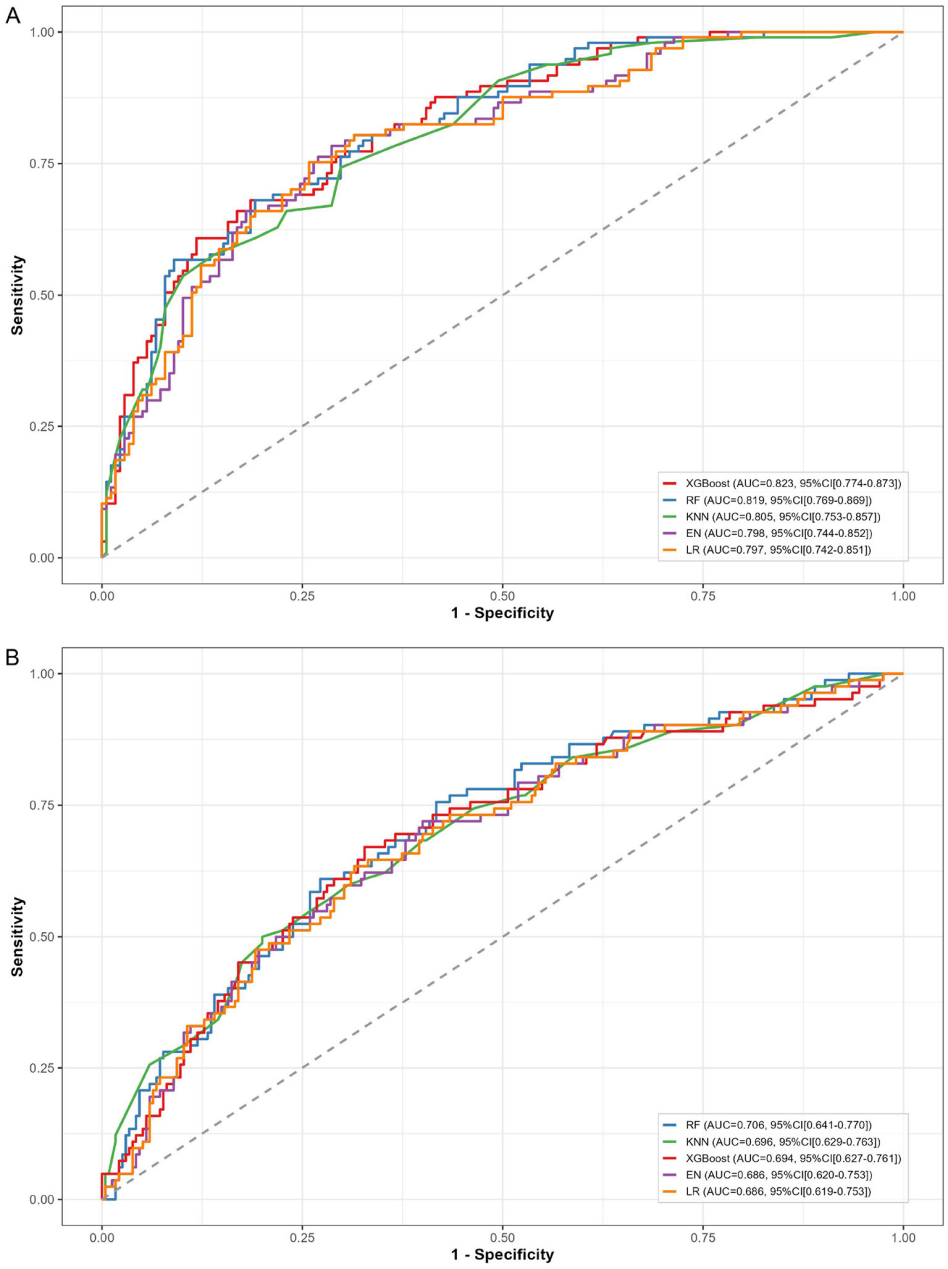
Receiver operating characteristic curves for all 5 models. **(A)** Internal test cohort; **(B)** External validation cohort. RF, random forest; KNN, K-nearest neighbors; XGB, extreme gradient boosting; EN, elastic net regression; LR, logistic regression.

Among the 5 ML algorithms evaluated, the RF model demonstrated the most balanced discriminative performance [Internal AUC: 0.819 (95% CI: 0.769–0.869); External AUC: 0.706 (95% CI: 0.641–0.770)] and acceptable generalization capacity (Figure 2). In comparison, the AUCs for the remaining 4 models ranged from 0.797 to 0.823 internally (with XGBoost achieving a marginally higher AUC) and from 0.686 to 0.696 externally.

Compared to other models, the RF model exhibited comparable classification metrics (Table 2). In the internal testing set, it achieved an accuracy of 0.764, a sensitivity of 0.680, a specificity of 0.809, a PPV of 0.660, and a NPV of 0.823. In the external validation set, while the overall accuracy (0.628) and PPV (0.388) decreased as expected, the sensitivity shifted to 0.756, and the model maintained a robust NPV of 0.873.

**Table 2:** Model performance metrics.

Model	Accuracy, mean (95% CI)	Sensitivity, mean (95% CI)	Specificity, mean (95% CI)	PPV, mean (95% CI)	NPV, mean (95% CI)
Internal testing set					
XGboost	0.767 (0.713–0.818)	0.680 (0.586–0.773)	0.815 (0.757–0.873)	0.667 (0.580–0.759)	0.824 (0.764–0.879)
RF	0.764 (0.709–0.811)	0.680 (0.586–0.773)	0.809 (0.749–0.863)	0.660 (0.570–0.748)	0.823 (0.760–0.878)
KNN	0.716 (0.662–0.771)	0.742 (0.651–0.826)	0.702 (0.629–0.768)	0.576 (0.488–0.658)	0.833 (0.770–0.892)
Elastic Net	0.738 (0.687–0.793)	0.784 (0.700–0.862)	0.713 (0.644–0.780)	0.598 (0.518–0.684)	0.858 (0.801–0.912)
Logistic	0.745 (0.691–0.800)	0.753 (0.667–0.839)	0.742 (0.674–0.806)	0.613 (0.530–0.700)	0.846 (0.785–0.900)
External validation set					
RF	0.628 (0.580–0.678)	0.756 (0.662–0.844)	0.583 (0.527–0.646)	0.388 (0.310–0.461)	0.873 (0.814–0.921)
KNN	0.722 (0.669–0.767)	0.500 (0.386–0.610)	0.800 (0.749–0.849)	0.466 (0.360–0.568)	0.821 (0.767–0.869)
XGboost	0.672 (0.618–0.719)	0.671 (0.564–0.768)	0.672 (0.612–0.730)	0.417 (0.331–0.496)	0.854 (0.802–0.902)
Elastic Net	0.631 (0.577–0.681)	0.720 (0.618–0.821)	0.600 (0.538–0.660)	0.386 (0.307–0.455)	0.860 (0.801–0.912)
Logistic	0.672 (0.618–0.719)	0.634 (0.526–0.732)	0.685 (0.625–0.742)	0.413 (0.322–0.489)	0.843 (0.787–0.894)

**Figure 3:**
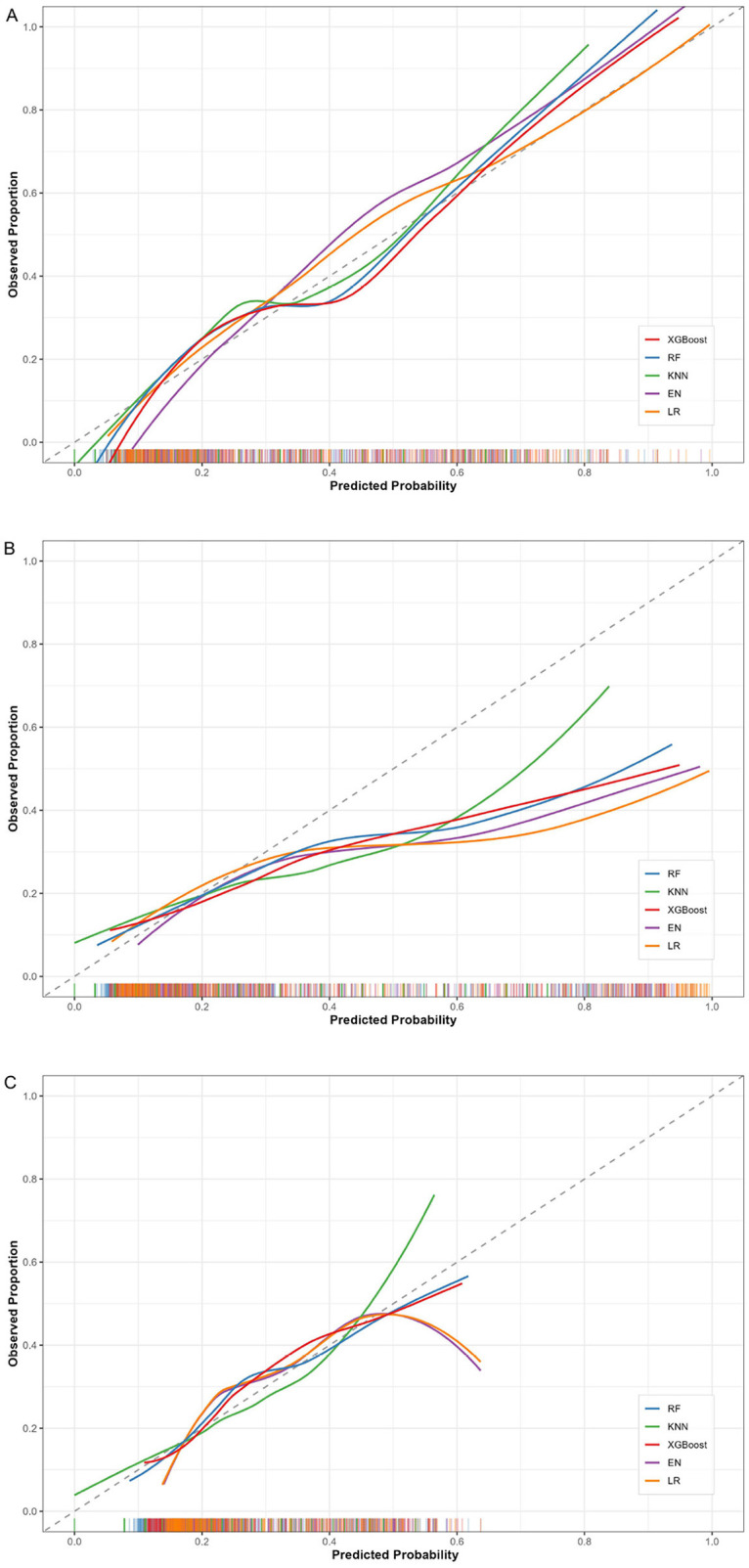
Calibration curves for all 5 models. **(A)** Internal test cohort; **(B)** external validation cohort before recalibration; **(C)** external validation cohort after recalibration. RF, random forest; KNN, K-nearest neighbors; XGB, extreme gradient boosting; EN, elastic net regression; LR, logistic regression.

Calibration curves (Figure 3) and detailed metrics (Table 3) indicated adequate internal calibration for most models (Hosmer–Lemeshow *p* > 0.05), except EN (*p* = 0.047). While initial external calibration was suboptimal across all models, logistic recalibration successfully restored performance, enabling all models to pass the test (*p* = 0.390–0.959). Specifically, the RF model demonstrated favorable internal fit (*p* = 0.592; ICI: 0.038; Brier score: 0.162). Following external recalibration, its performance adjusted accordingly: the *p*-value increased from < 0.001 to 0.959, with the ICI and Brier score optimizing to 0.018 and 0.173, respectively. Ultimately, the recalibrated RF model provides more appropriate delirium risk estimates in independent data.

**Table 3:** Model caliberation metrics.

Model	Hosmer–Lemeshow, *P* value	ICI, mean	Brier score, mean
Internal testing set			
XGboost	0.382	0.038	0.162
Random Forest	0.592	0.038	0.162
KNN	0.429	0.042	0.170
Elastic Net	0.047	0.051	0.172
Logistic	0.057	0.025	0.170
External validation set (before recaliberation)			
Random Forest	<0.001	0.118	0.206
KNN	<0.001	0.100	0.189
XGboost	<0.001	0.130	0.211
Elastic Net	<0.001	0.136	0.224
Logistic	<0.001	0.162	0.241
External validation set (after recaliberation)			
Random Forest	0.959	0.018	0.173
KNN	0.503	0.020	0.174
XGboost	0.390	0.018	0.174
Elastic Net	0.921	0.031	0.177
Logistic	0.761	0.032	0.177

**Figure 4:**
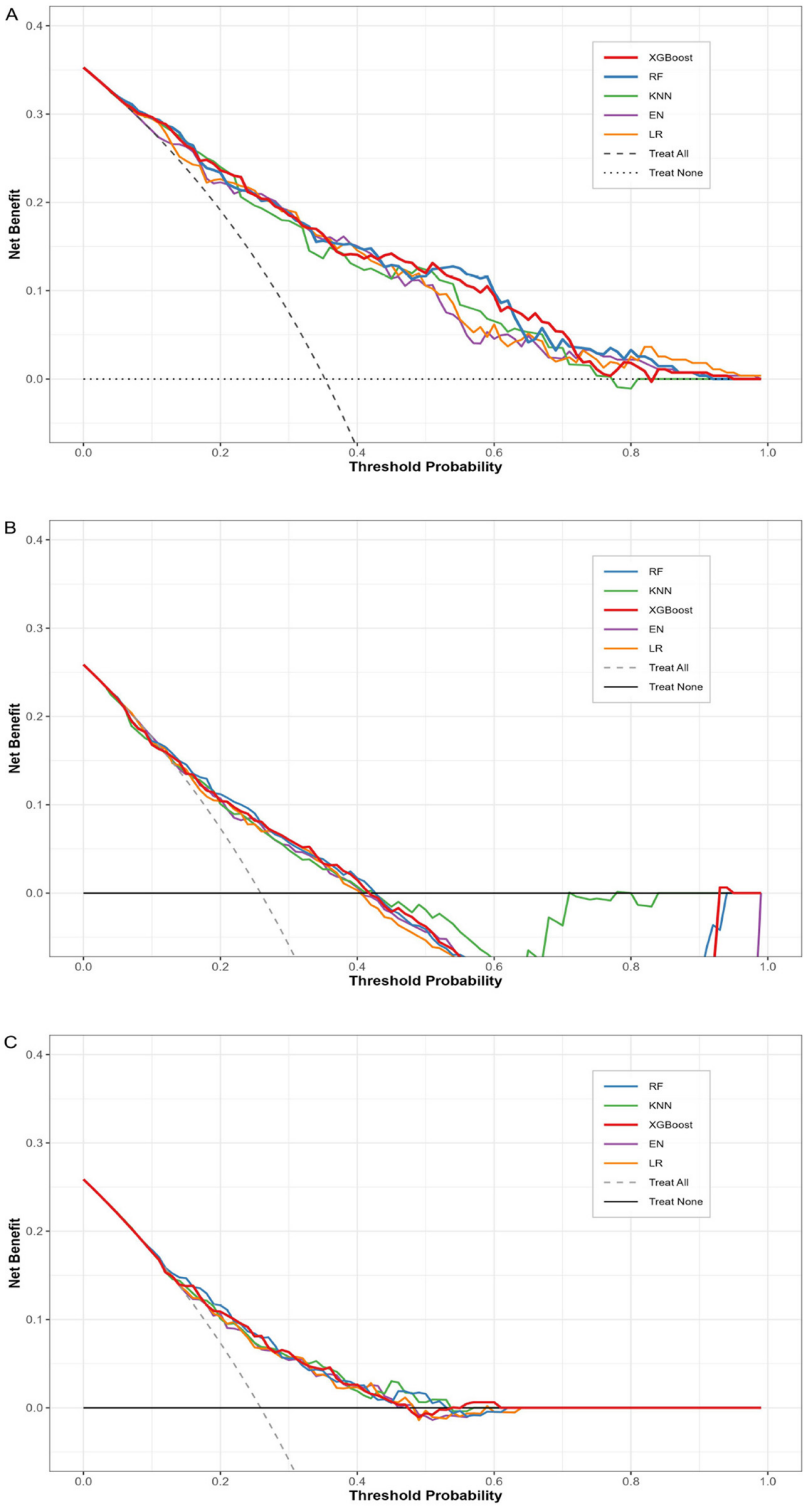
DCA for all 5 models. **(A)** Internal test cohort; **(B)** External validation cohort before recalibration; **(C)** External validation cohort after recalibration. RF, random forest; KNN, K-nearest neighbors; XGB, extreme gradient boosting; EN, elastic net regression; LR, logistic regression.

Decision curve analysis (DCA) further confirmed the clinical utility of the RF model (Figure 4). Internally, it produced positive net benefits across a wide threshold range (0.03–0.91), achieving a maximum added benefit of 0.155. In the external validation set (after recalibration), the RF model retained positive clinical benefit with a maximum added benefit of 0.080 and an effective threshold coverage between 0.09 and 0.53. The DCA parameters in detail were illustrated in Supplementary Table 2.

In summary, the RF model was selected as the primary predictive model in this study due to its balanced discriminative capacity, adaptability to recalibration, and retained clinical utility.

### 3.4 Model interpretability

**Figure 5:**
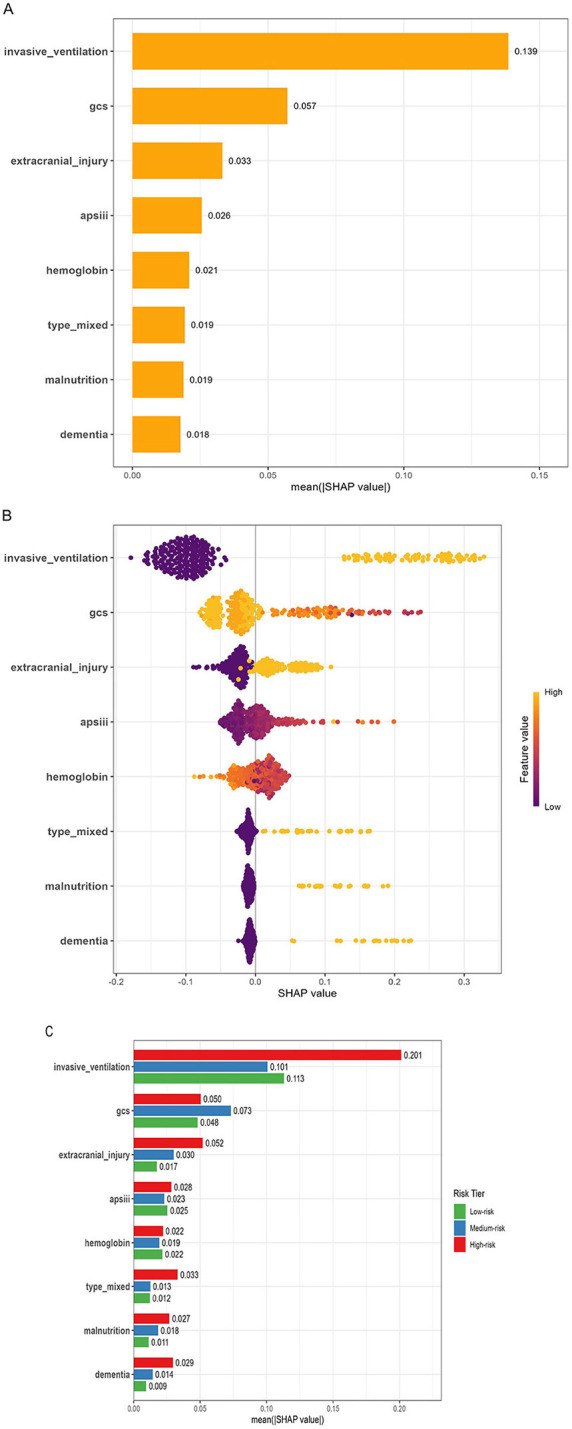
Interpretation of the prediction model using SHAP analysis. **(A)** Feature importance ranking: The greater the variable importance, the longer the corresponding bar; **(B)** Beeswarm plot: Yellow dots indicate high feature values, purple dots indicate low feature values, and the horizontal spread reflects the direction of the feature's impact. **(C)** Stratified SHAP feature importance by risk tier (low-, medium-, and high-risk groups defined by tertiles of predicted probability). GCS, Glasgow coma scale; APSIII, acute physiology score III.

SHAP analysis was employed to visualize predictor contributions (Figure 5A). Invasive ventilation emerged as the most predominant predictor of the outcome, followed by the Glasgow Coma Scale (GCS) score and extracranial injury. As indicated by the summary plot (Figure 5B), the presence of invasive ventilation, extracranial injury, and mixed intra-/extra-axial injury, along with higher APSIII scores, were associated with positive SHAP values, contributing to an increased risk of the outcome. Conversely, GCS scores and hemoglobin levels exhibited an inverse relationship with the outcome; higher values of these features (represented by yellow dots) were associated with negative SHAP values, suggesting a protective effect. Notably, the feature importance of invasive ventilation [mean |SHAP value| = 0.139] was substantially higher than all other variables in the model.

Stratified SHAP analysis (Figure 5C) revealed distinct patterns of feature importance across different risk tiers. Specifically, the clinical decision cutoffs for the low-, medium-, and high-risk tiers were defined based on the tertiles of the predicted probabilities as 0.029–0.165, 0.165–0.439, and >0.439, respectively. For patients in the high-risk group, invasive ventilation emerged as the overwhelming driver of delirium risk, with a mean absolute SHAP value of 0.201, significantly higher than its contribution in the medium-risk (0.101) or low-risk (0.113) groups. Conversely, the Glasgow Coma Scale (GCS) score demonstrated its peak predictive sensitivity in the medium-risk tier (0.073), compared to 0.050 and 0.048 in the high- and low-risk tiers, respectively. Other anatomical and physiological factors, such as extracranial injury, mixed intra-/extra-axial injury, and malnutrition, also exhibited a progressive increase in their risk-driving impact from the low-risk to the high-risk stratum.

**Figure 6:**
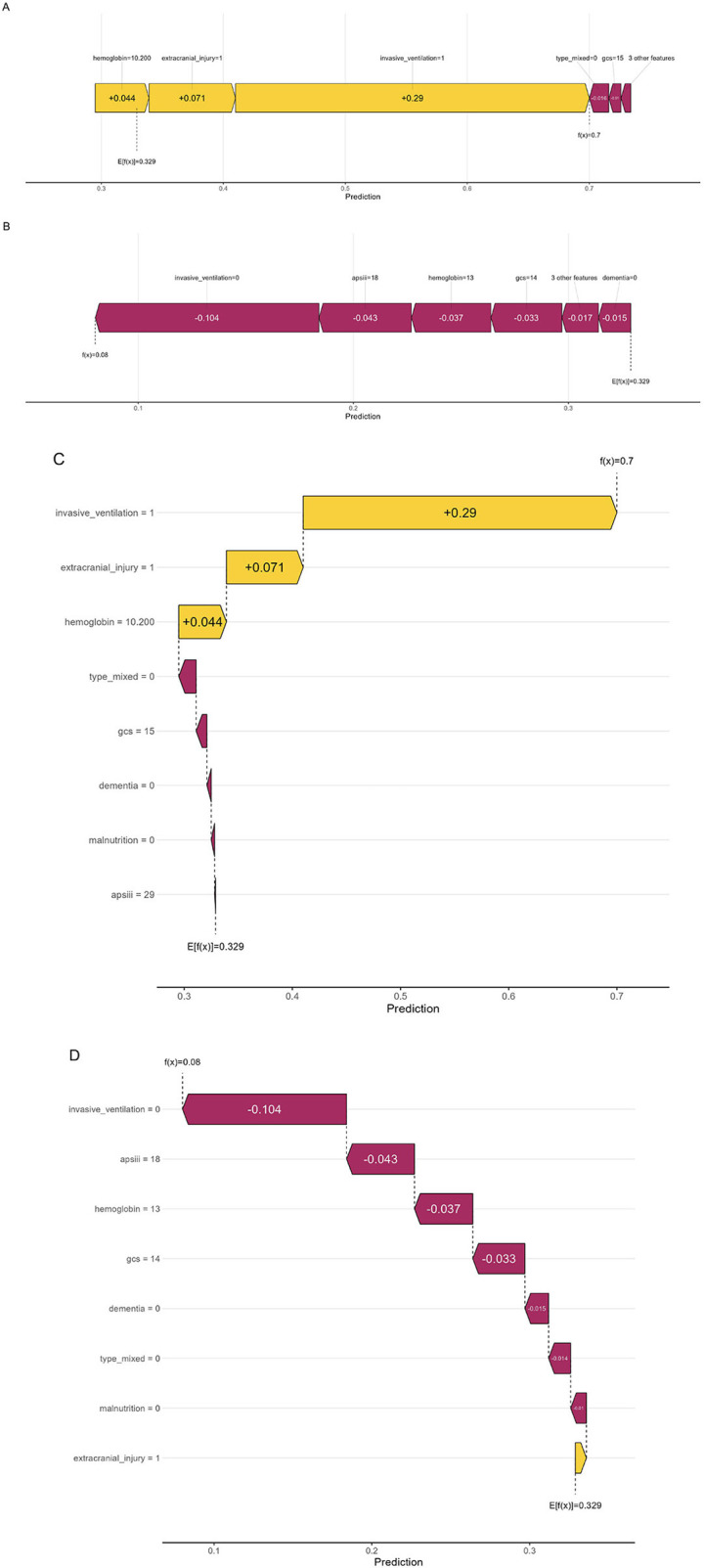
Visual interpretation of risk factors for two representative patient cases based on SHAP. **(A)** Force plot with delirium; **(B)** Force plot without delirium; **(C)** Waterfall plot with delirium; **(D)** Waterfall plot without delirium. Yellow indicates a positive impact on the occurrence of delirium, and red indicates a negative impact. The bar length represents the magnitude of the contribution. GCS, Glasgow coma scale; APSIII, acute physiology score III.

We utilized SHAP force and waterfall plots to visualize individual predictions for two representative cases, with colors denoting varying feature contributions (Figure 6). Figure 6A depicts a patient with a high predicted risk. In this case, the use of invasive ventilation (=1), the presence of an extracranial injury (=1), and a low hemoglobin level (=10.20) exerted significant positive impacts on the outcome (yellow bars), thereby increasing the risk. Conversely, a GCS score of 15 and the absence of a mixed intra-/extra-axial injury (type_mixed = 0) showed minor negative impacts (purple bars). The model's output value was 0.700, which is substantially higher than the baseline value of 0.329. In this instance, the total positive contributions significantly exceeded the negative ones, leading the model to predict a high probability of the outcome. Figure 6B illustrates a patient with a low predicted risk. Although the presence of extracranial injury (=1) had a slight positive driving effect on the outcome, the lack of invasive ventilation (=0), a lower APSIII score (=18), a GCS score of 14, a normal hemoglobin level (=13.00), and the absence of a mixed intra-/extra-axial injury (type_mixed = 0) exhibited primary negative impacts, significantly reducing the probability of the outcome. The model's output value was 0.080, which is well below the baseline value of 0.329. In this scenario, the negative contributions (purple bars) heavily predominated. Figures 6C, D present waterfall plots for the aforementioned cases as an alternative visualization of the SHAP values. From these graphs, it is evident that invasive ventilation, extracranial injury, GCS score, hemoglobin levels, and mixed intra-/extra-axial injury are the most crucial patient-specific variables driving the model's individual predictions.

### 3.5 Sensitivity analysis

After reclassifying deceased patients who had not developed delirium as delirium-positive, the incidence of delirium increased slightly (Internal: 36.00%; External: 26.81%), yet discriminative performance remained stable. The RF model achieved an AUC of 0.825 (95% CI: 0.776–0.874) in the internal cohort and 0.716 (95% CI: 0.653–0.779) in the external cohort (Supplementary Figure 3). DeLong tests confirmed no statistically significant differences in AUCs compared to the primary analysis (*p* = 0.867 and *p* = 0.822, respectively). Similarly, when MICE was employed as an alternative to random forest-based imputation, no significant deviations in AUC were observed for any of the five algorithms in either cohort (all *p* > 0.90 in DeLong tests). Specifically, the primary RF model maintained an AUC of 0.820 internally and 0.702 externally (Supplementary Figure 4).

### 3.6 Subgroup analysis

Based on the SHAP analysis, we selected the top three predictors (invasive ventilation, GCS score and extracranial injury) for detailed subgroup analyses. Additionally, age was included as a stratification factor due to its established clinical significance as a key predictor of delirium (29). In the subgroup analyses (Supplementary Table 3), the RF model demonstrated moderate discriminatory performance across clinically stratified subgroups. Within the internal test set, its discriminative ability declined in patients with higher clinical severity (e.g., those with GCS ≤ 12 or invasive ventilation). This trend became more pronounced in the external validation set, where the AUC for these severe subgroups dropped to 0.578–0.589. In contrast, the model showed better generalizability in younger patients (AUC = 0.780) and in those with extracranial injuries (AUC = 0.762).

## 4 Discussion

In this study, we developed and validated early predictive models for incident delirium in patients with TBI. Among the five ML algorithms trained on the MIMIC-IV dataset, the RF model demonstrated the most favorable discriminative ability during internal validation (AUC = 0.819), alongside adequate calibration and clinical net benefit. When applied to the external eICU-CRD dataset, the model maintained acceptable generalizability, though with an expected reduction in performance (AUC = 0.706).

The observed attenuation in the model's performance upon external validation is expected and aligns with the known challenge of generalizing predictive models across heterogeneous clinical settings. This is illustrated by the PRE-DELIRIC model, whose reported AUCs vary considerably (0.60–0.84) across different cohorts (10–13). The primary driver of this performance shift in our study is the inherent clinical and institutional heterogeneity of the eICU-CRD database, which aggregates data from 208 distinct hospitals. This diversity is reflected in differing baseline characteristics and delirium incidence rates between our development (35.27%) and validation (25.86%) cohorts. Importantly, our observed performance shift is consistent with previous research, which indicates that ML models for delirium typically experience an AUC decline of 8.86% to 17.58% when applied to heterogeneous external cohorts (18, 21, 30–32). Thus, while highlighting the ongoing challenge of model generalization, the RF model's performance remains within a reasonable and realistic range for diverse real-world populations.

Subgroup analyses further elucidate the performance degradation, highlighting how baseline disease severity interacts with multicenter heterogeneity. Within the internal cohort, the model showed reduced discriminative capacity for the most severely ill patients (e.g., GCS ≤ 12 or requiring invasive ventilation), likely due to a “range restriction” effect where uniformly severe physiological derangements limit the predictive variance of static features (33). In the multicenter external setting, this limitation is exacerbated by heterogeneous clinical management (e.g., sedation protocols) (34), which attenuates the prognostic value of initial 24-h data, leading to lower external AUCs in these subgroups. Conversely, the model generalized better in younger patients and those with extracranial injuries, phenotypes that likely present more consistent physiological signals across institutions. These findings define the model's clinical boundaries: it serves as a useful screening tool for younger or less severely ill patients, whereas predicting delirium in the elderly or those with high baseline severity may require incorporating dynamic, institution-specific treatment variables (35).

Despite this expected attenuation, the RF model's potential for clinical translation is supported by several practical strengths. First, it maintained a high negative predictive value (NPV of 0.873) in the external cohort, suggesting its value as a potential “rule-out” tool to help avoid unnecessary interventions in low-risk patients, thereby aiding in resource allocation (36). Second, although external data heterogeneity initially impacted calibration, standard logistic recalibration successfully restored its fit (Hosmer–Lemeshow *p* = 0.959). This demonstrates the model's underlying adaptability (37), indicating that reliable risk estimation can be achieved in new environments following simple baseline adjustments. Third, decision curve analysis indicated positive net clinical benefits (0.155 and 0.080) across a clinically relevant range of threshold probabilities, demonstrating its practical value in clinical settings (38). Finally, the model maintained consistent performance throughout the sensitivity analyses. Its discriminative ability was not substantially affected when accounting for the competing risk of early mortality, and its predictive accuracy remained comparable between the missForest and MICE imputation strategies. This consistency under varying methodological assumptions supports the model's potential utility in complex severe TBI environments.

By leveraging the SHAP approach, we enhanced the interpretability of our model, overcoming the “black-box” limitation often associated with advanced algorithms. At the global level, our SHAP analysis identified invasive ventilation as the most predominant driver of delirium risk. This finding is clinically grounded in the dual mechanism of invasive ventilation: it serves as a proxy for critical respiratory or neurological failure, while simultaneously introducing iatrogenic precipitants, including immobilization, sensory deprivation, and the consequent need for sedation, all of which are established triggers for ICU delirium (39). Following this, the prominence of GCS, extracranial injury, and APSIII aligns with the “vulnerable brain” hypothesis, where direct neuronal damage and systemic inflammatory stress lower the threshold for acute brain dysfunction (40, 41). Furthermore, our global analysis consistently demonstrated that lower hemoglobin (Hb) levels are associated with an increased delirium risk. From a pathophysiological perspective, cerebral hypoxia resulting from anemia may impair the synthesis of oxygen-sensitive neurotransmitters and exacerbate oxidative stress (42). Therefore, maintaining optimal Hb targets represents a modifiable, foundational neuroprotective strategy to mitigate metabolic stress across all TBI patients.

However, global feature importance often lacks the granularity needed for bedside application. To facilitate clinical implementation, we defined three risk tiers based on the tertiles of predicted probabilities, pairing each stratum with targeted monitoring and intervention strategies.

Within the high-risk tier, the impact of invasive ventilation is most pronounced. This explains why varying sedation protocols across eICU-CRD hospitals affected our model's external performance for intubated patients. Clinically, this highlights the need for targeted “light sedation” and early weaning strategies to facilitate cognitive assessment (43, 44).

For the intermediate-risk tier, GCS scores exhibited their highest relative predictive importance. Consequently, a low or fluctuating GCS score, particularly when observed alongside a high APSIII and complex primary injuries, should trigger closer neurological monitoring (e.g., more frequent CAM-ICU screenings). The clinical focus in this scenario is to promptly address the underlying causes without unnecessarily escalating sedative use.

Finally, the low-risk tier is characterized by the absence of dominant acute precipitants. Here, the model serves as a “rule-out” tool, supporting a “watchful waiting” approach. Identifying these patients assures clinicians that foundational care, such as optimizing Hb targets, is sufficient, thereby preventing the over-utilization of sedatives and monitoring resources.

Unlike traditional regression coefficients that provide population-level averages, SHAP force plots allow clinicians to decompose the risk for an individual patient. For instance, our individualized analysis demonstrated how the absence of invasive ventilation, a lower APSIII score, and the lack of a mixed intra-/extra-axial injury can drive the specific risk prediction down, even in the presence of anatomical trauma such as an extracranial injury. This level of interpretability supports the shift toward precision medicine, enabling early, targeted, and individualized interventions for patients (45).

This study has several limitations. First, the reliance on intermittent retrospective clinical charting introduces a risk of underdiagnosing transient delirious episodes, potentially leading to outcome capture heterogeneity across diverse healthcare systems. Second, the exclusive use of static clinical variables from the initial 24-h window fails to capture dynamic pathophysiological trajectories and consequently restricts the model's generalizability across institutions with varying management protocols. Third, due to the constraints of the public databases, detailed neuroimaging data could not be included, which is a significant limitation of this study and may affect the comprehensive assessment of delirium risk. Furthermore, framing early delirium prediction as a fixed-window binary classification task structurally precluded formal time-to-event competing risk methods (e.g., the Fine-Gray model). Although we mitigated this statically via sensitivity analysis (reclassification), dynamic modeling of competing early mortality remains an important direction for future refinement.

## 5 Conclusion

This study developed and evaluated a ML-based predictive model for incident delirium in patients with TBI. While the RF model showed the expected decline in performance during multicenter external validation, it retained acceptable discriminative ability and clinical net benefit. Supported by SHAP analysis, this model provided a three-tiered risk stratification framework to guide graded interventions, serving as a valuable supplementary tool to assist in early risk stratification within the neurocritical care setting.

## Data Availability

Publicly available datasets were analyzed in this study. This data can be found here: 1. MIMIC-IV (Medical Information Mart for Intensive Care-IV) Repository Name: PhysioNet Direct Link: https://physionet.org/content/mimiciv/ Accession Number (DOI): 10.13026/kpb9-mt58 (Version 3.1) Project Website: https://mimic.mit.edu 2. eICU-CRD (eICU Collaborative Research Database) Repository Name: PhysioNet Direct Link: https://physionet.org/content/eicu-crd/ Accession Number (DOI): 10.13026/C2WM1R (Version 2.0) Project Website: https://eicu-crd.mit.edu.
