# Supplementary material for: Early prediction of incident delirium in traumatic brain injury: a multicenter validated and interpretable machine learning approach
Source: Front Neurol. 2026 May 21;17:1848730. doi: 10.3389/fneur.2026.1848730 (PMC13235148; doi:10.3389/fneur.2026.1848730)
Supplement: Supplementary file 1 [file Supplementary_file_1.docx]

# Early Prediction of Incident Delirium in Traumatic Brain Injury: A Multicenter Validated and Interpretable Machine Learning Approach

Correspondence: Jianchao Liu, 13264006725@163.com；Lihua Liu, Liulihua07@yeah.net.

**Supplemental Table 1 The parameters of model development**

| **Algorithm** | **Parameters** |
| --- | --- |
| LR | method='glm', family='binomial', metric='ROC', preProcess=c('center', 'scale'), trControl=10-fold CV. |
| EN | method='glmnet', metric='ROC', preProcess=c('center', 'scale'), trControl=10-fold CV. Hyperparameter Search Grid: alpha=seq(0.1, 0.9, length.out=10), lambda=10^seq(-5, 1, length.out=30). |
| RF | method='ranger', metric='ROC', num.trees=1000, importance='permutation', trControl=10-fold CV. Hyperparameter Search Grid: mtry={3, 4, 5}, splitrule={'gini', 'extratrees'}, min.node.size={5, 10, 15}. |
| KNN | method='knn', metric='ROC', preProcess=c('center', 'scale'), trControl=10-fold CV. Hyperparameter Search Grid: k={3, 5, 7, 11, 15, 21, 31, 41}. |
| XGBoost | package='xgboost', objective='binary:logistic', eval_metric='auc', eta=0.02, nrounds=Up to 2000 (with early stopping=50). Random Search (80 iterations) Space: max_depth={3, 4, 5}, min_child_weight={1, 5, 10}, gamma={0, 1, 3, 5}, subsample={0.6, 0.7, 0.8}, colsample_bytree={0.6, 0.7, 0.8}, colsample_bynode={0.6, 0.7, 0.8}, reg_alpha={0, 1, 5}, reg_lambda={0, 1, 5}. |

**Supplemental Table 2 DCA parameters**

| **Model** | **Minimal Threshold** | **Maximum Threshold** | **Max Added Benefit** | **Optimal Threshold** |
| --- | --- | --- | --- | --- |
| **Internal testing set** |  |  |  |  |
| XGboost | 0.05 | 0.94 | 0.159 | 0.35 |
| Random Forest | 0.03 | 0.91 | 0.155 | 0.36 |
| KNN | 0.01 | 0.77 | 0.149 | 0.36 |
| Elastic Net | 0.10 | 0.98 | 0.161 | 0.38 |
| Logistic | 0.06 | 0.99 | 0.158 | 0.36 |
| **External validation set (before recaliberation)** | | | | |
| Random Forest | 0.04 | 0.42 | 0.079 | 0.25 |
| KNN | 0.01 | 0.79 | 0.071 | 0.26 |
| XGboost | 0.12 | 0.94 | 0.081 | 0.26 |
| Elastic Net | 0.10 | 0.40 | 0.077 | 0.26 |
| Logistic | 0.06 | 0.40 | 0.070 | 0.27 |
| **External validation set (after recaliberation)** | | | | |
| Random Forest | 0.09 | 0.53 | 0.080 | 0.27 |
| KNN | 0.01 | 0.53 | 0.069 | 0.26 |
| XGboost | 0.11 | 0.60 | 0.081 | 0.26 |
| Elastic Net | 0.14 | 0.59 | 0.066 | 0.26 |
| Logistic | 0.14 | 0.59 | 0.067 | 0.27 |

**Supplemental Table 3 Subgroup analysis of the RF model in internal testing and external validation Cohorts**

| **Subgroup Variables** | **Internal Testing Set (N = 275)** | | **External Validation Set (N = 317)** | |
| --- | --- | --- | --- | --- |
|  | **N** | **AUC (95% CI)** | **N** | **AUC (95% CI)** |
| **GCS Score** |  |  |  |  |
| ≤12 (Moderate/Severe TBI) | 41 | 0.720  (0.560 - 0.880) | 116 | 0.578  (0.470 - 0.686) |
| > 12 (Mild TBI) | 234 | 0.834  (0.781 - 0.887) | 201 | 0.652  (0.546 - 0.758) |
| **Invasive Ventilation** |  |  |  |  |
| Yes | 81 | 0.712  (0.594 - 0.831) | 95 | 0.589  (0.474 - 0.705) |
| No | 194 | 0.764  (0.691 - 0.837) | 222 | 0.640  (0.546 - 0.734) |
| **Age (years)** |  |  |  |  |
| ≥65 | 155 | 0.761  (0.684 - 0.837) | 149 | 0.611  (0.503 - 0.719) |
| < 65 | 120 | 0.891  (0.834 - 0.948) | 168 | 0.780  (0.707 - 0.854) |
| **Extracranial Injury** |  |  |  |  |
| Yes | 119 | 0.826  (0.752 - 0.901) | 84 | 0.762  (0.650 - 0.874) |
| No | 156 | 0.760  (0.681 - 0.839) | 233 | 0.690  (0.613 - 0.767) |

**
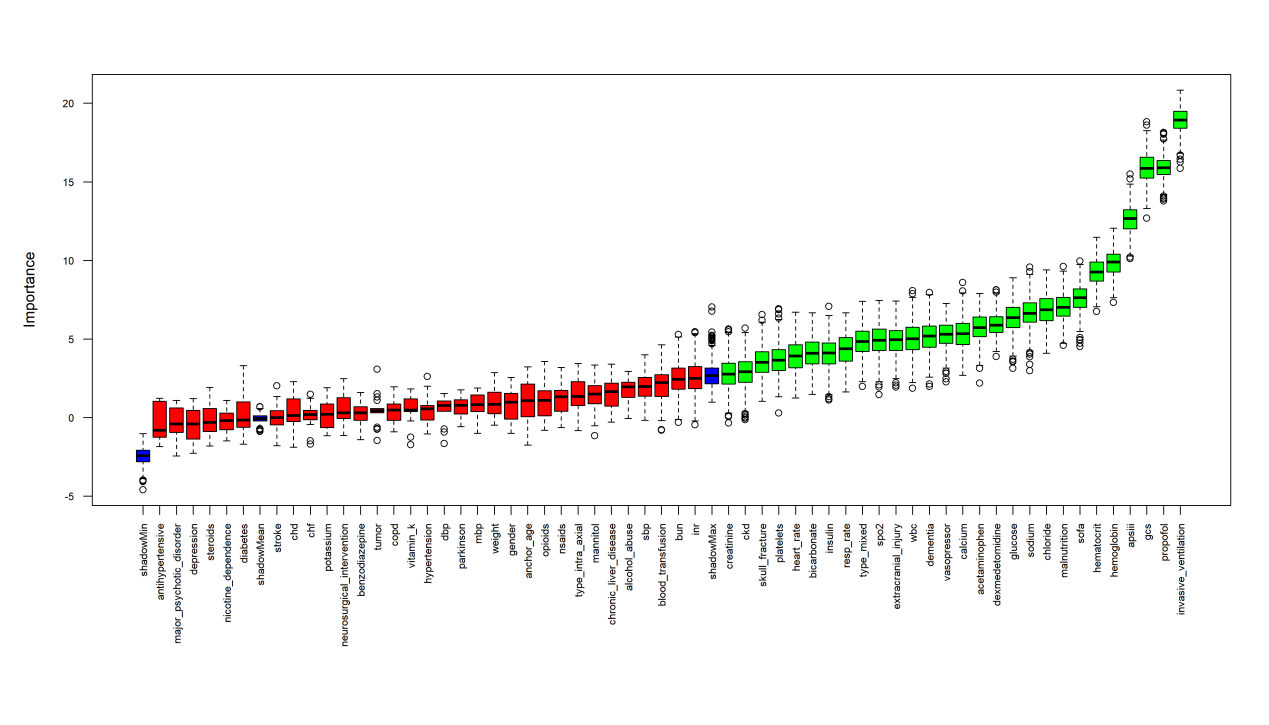
**

**Supplemental Figure 1**

**Feature selection and importance ranking based on the Boruta algorithm**

**
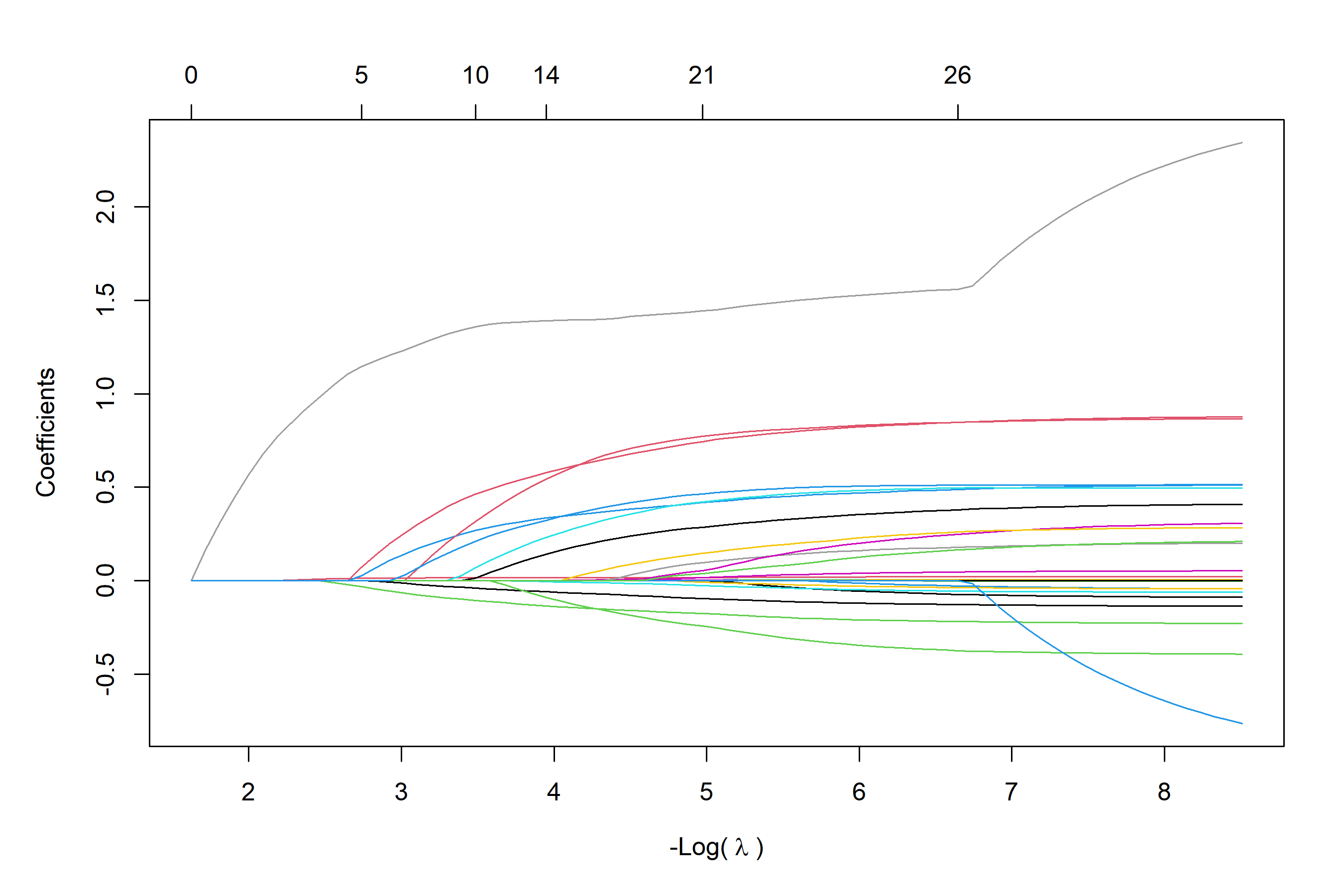

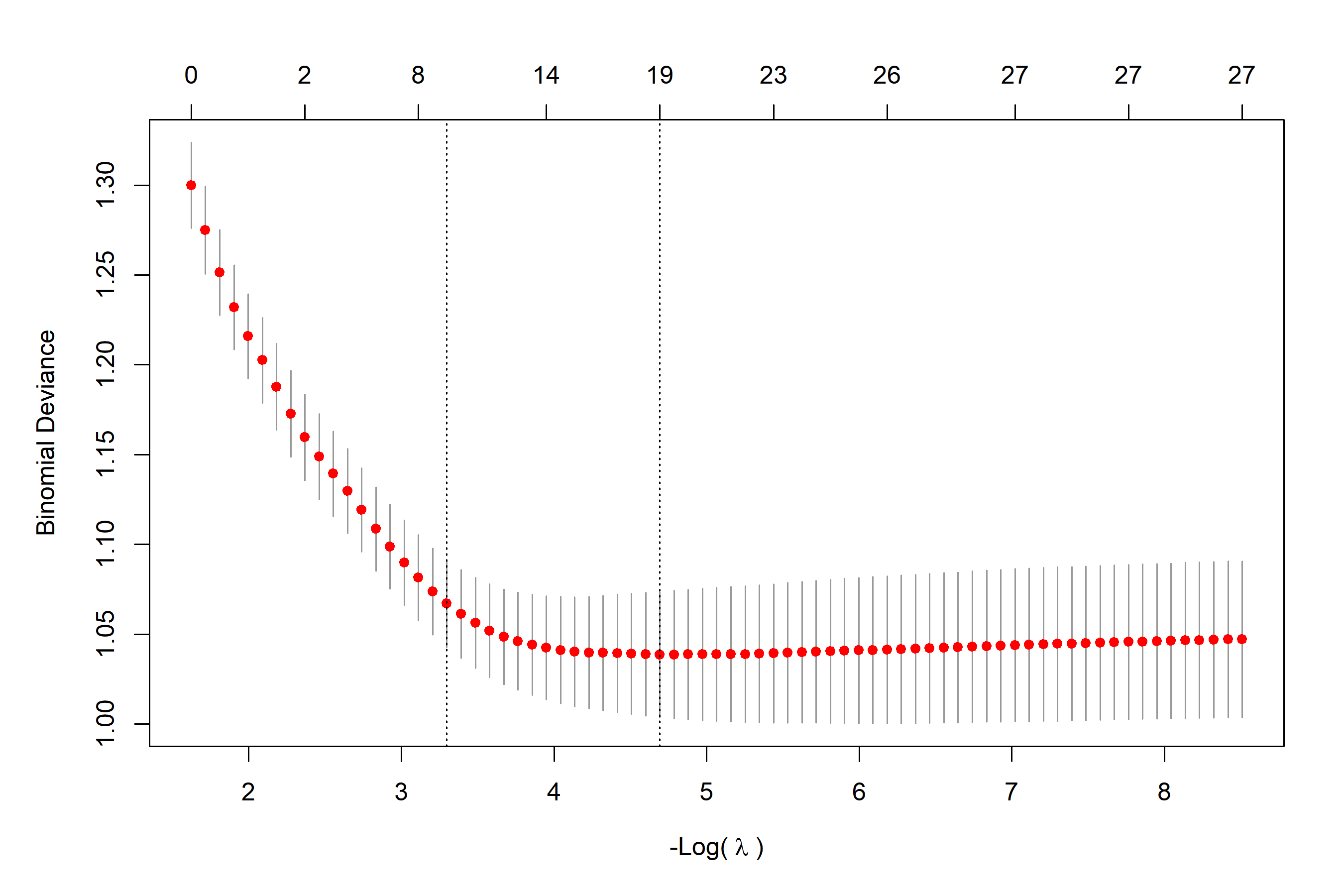
**

**Supplemental Figure 2**

**Feature selection and importance refining based on the LASSO regression**

A: LASSO coefficient profiles of the candidate features; B: Selection of the optimal tuning parameter λ using ten-fold cross-validation.


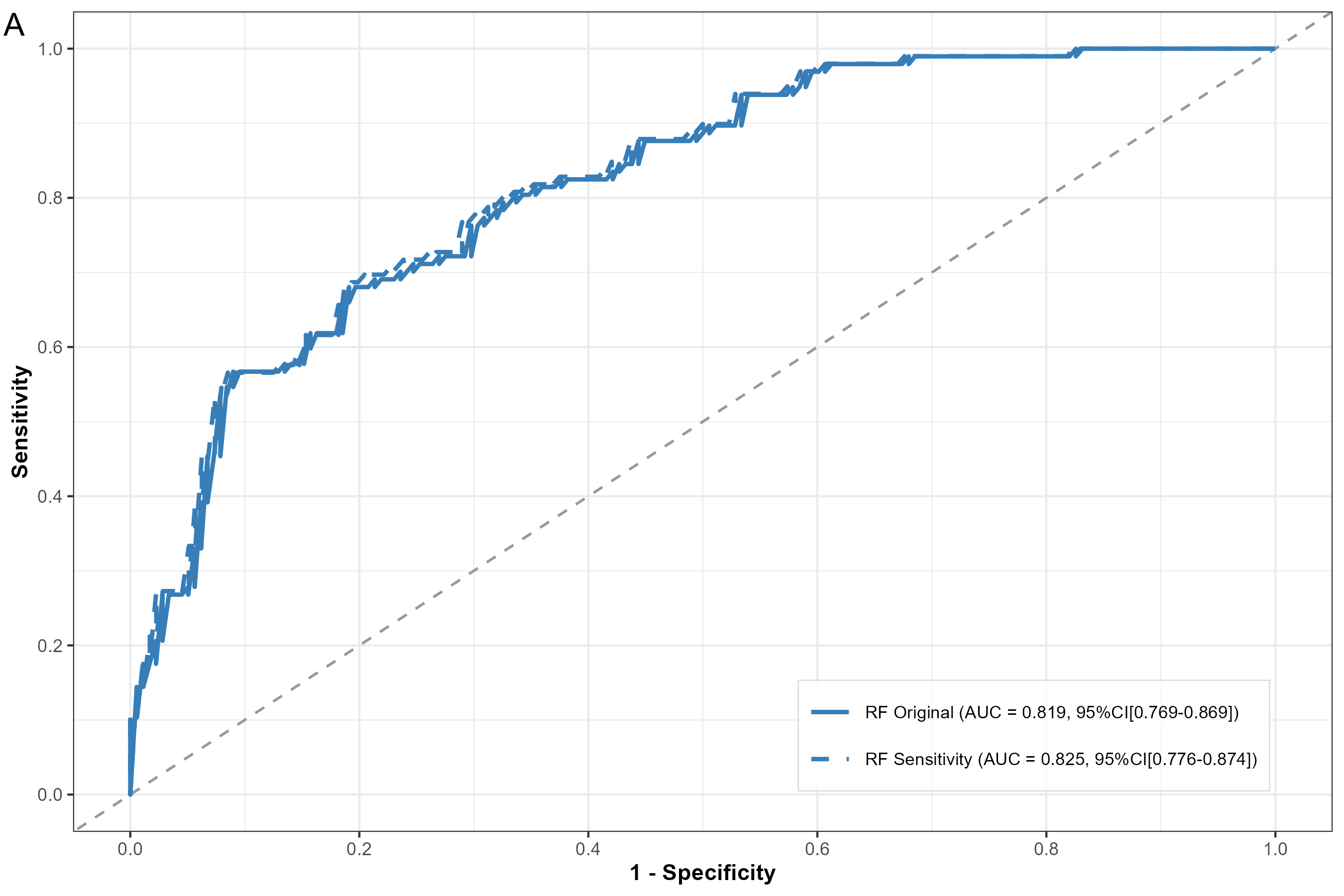


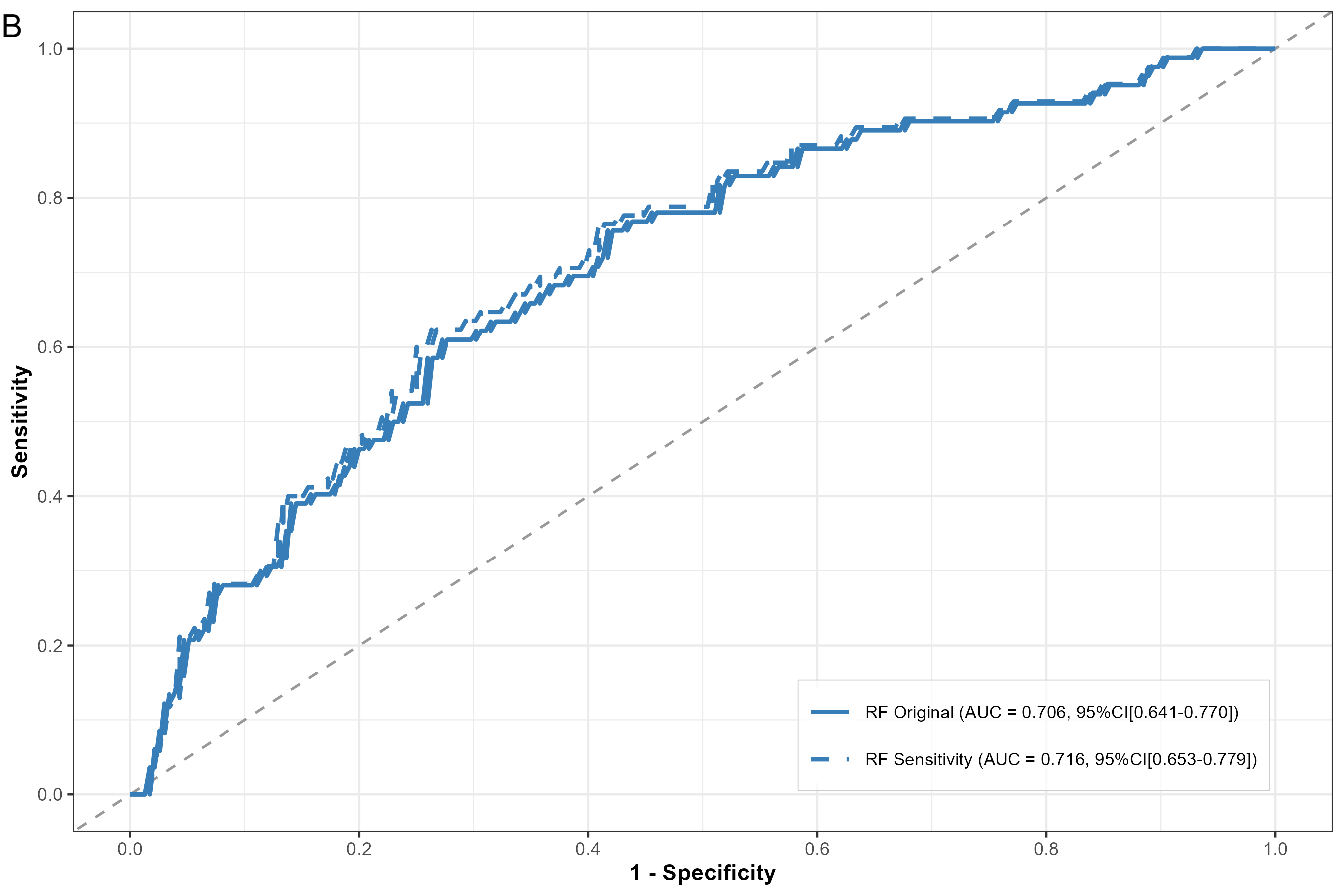


**Supplemental Figure 3**

**Sensitivity analysis accounting for the competing risk of death**

**(A)** Internal test cohort; **(B)** External validation cohort.

**
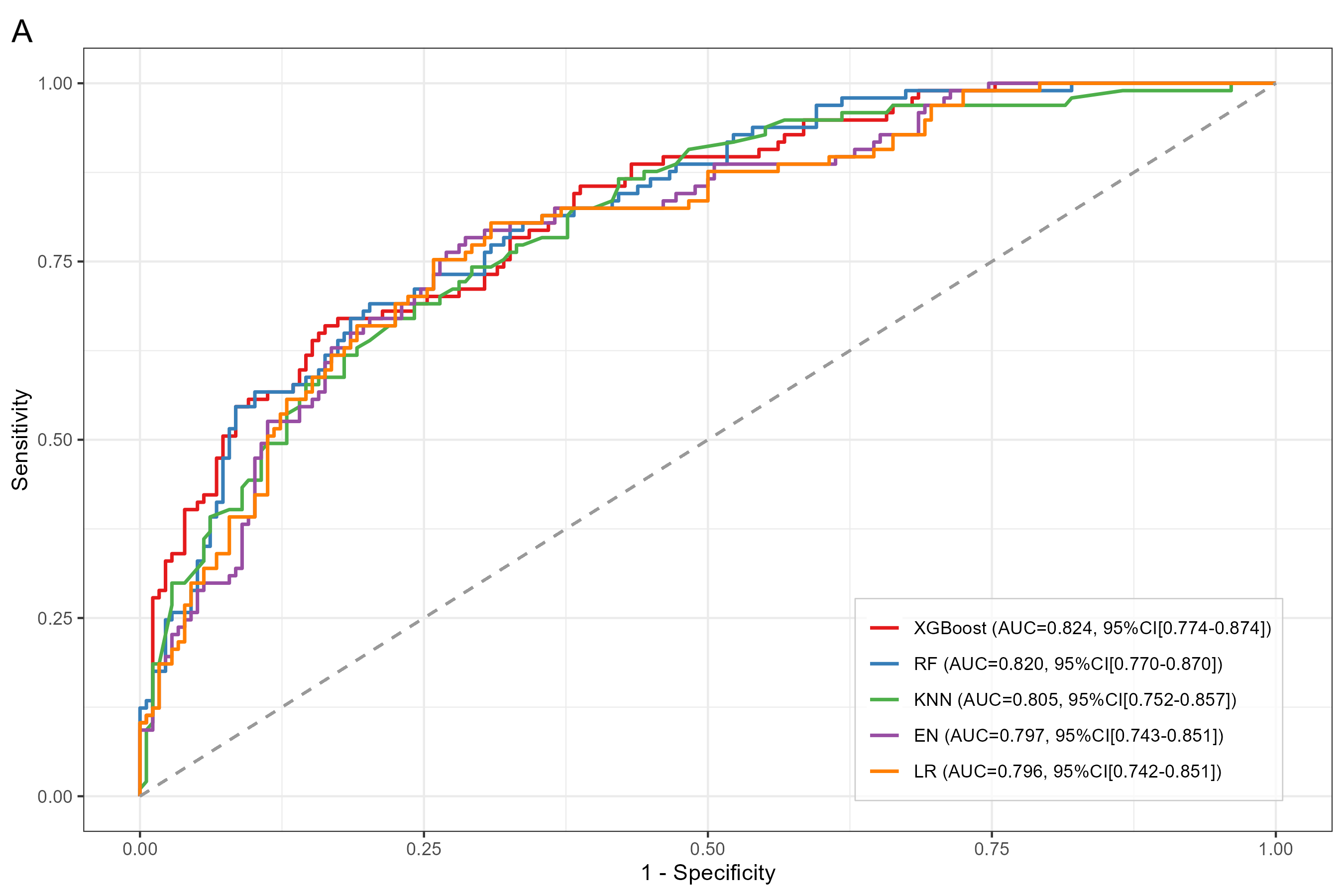
**

**
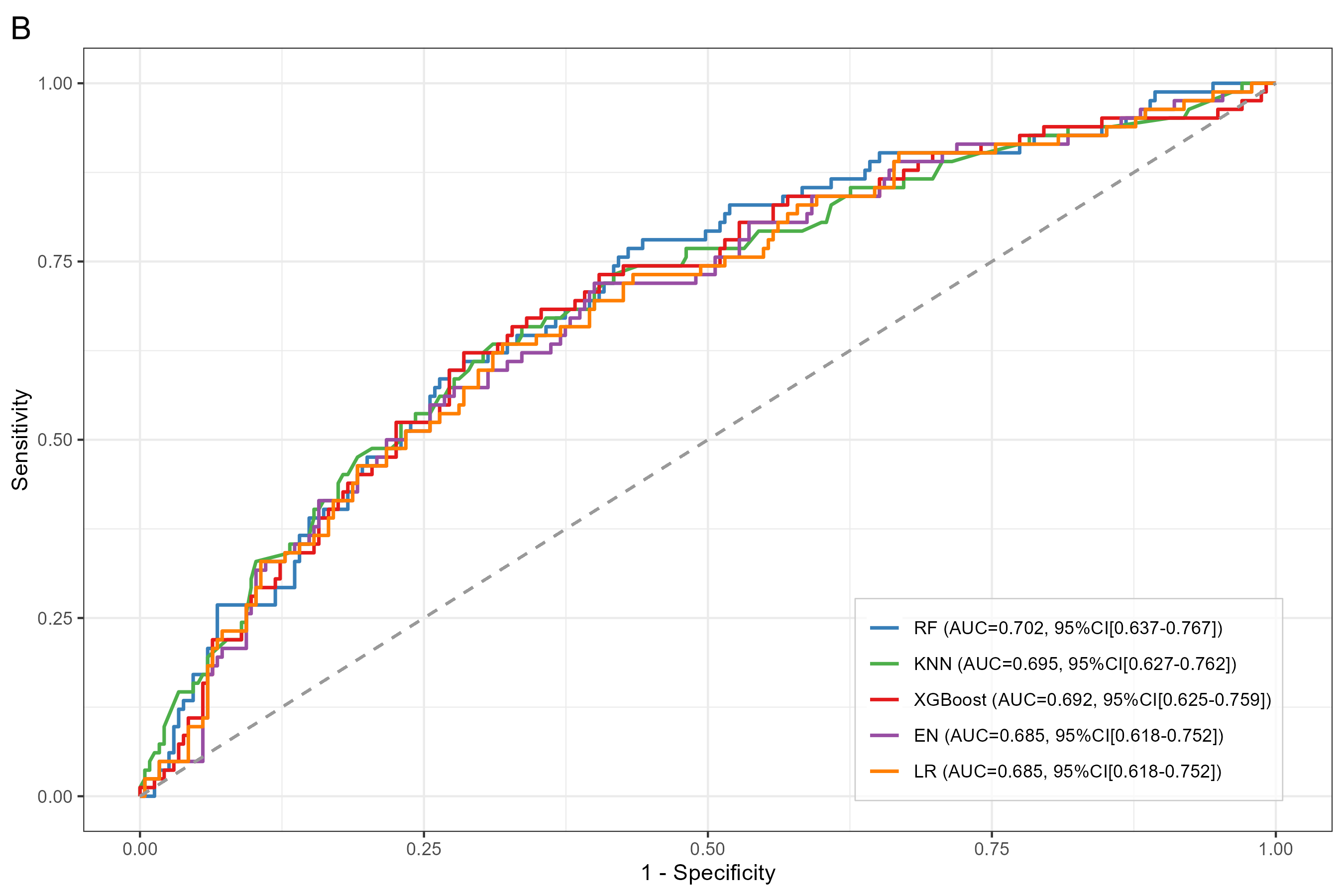
**

**Supplemental Figure 4**

**Sensitivity analysis using Multiple Imputation by Chained Equations (MICE) for missing data**

**(A)** Internal test cohort; **(B)** External validation cohort.
